# Thoracoscopic Reconstruction with the Non-Flap Tunnel Technique for Esophagogastric Junction Cancer Requiring High Intramediastinal Anastomosis: A Case Report

**DOI:** 10.70352/scrj.cr.26-0472

**Published:** 2026-08-25

**Authors:** Takeshi Ono, Shun Miyasaka, Takano Ohta, Keigo Hayashi, Tomofumi Orokawa, Hiroki Sunagawa, Shigeru Lee

**Affiliations:** Department of Surgery, Nakagami Hospital, Okinawa, Okinawa, Japan

**Keywords:** esophagogastric junction cancer, non-flap tunnel technique, thoracoscopic reconstruction, intramediastinal anastomosis, proximal gastrectomy

## Abstract

**INTRODUCTION:**

The double-flap technique (DFT) is an established anti-reflux reconstruction method after proximal gastrectomy (PG) and may be advantageous for mediastinal reconstruction because of its low incidence of anastomotic leakage. However, thoracoscopic DFT reconstruction remains technically demanding because flap-related procedures increase procedural complexity. The non-flap tunnel technique (NFTT) has recently been introduced as a simplified alternative to DFT, but its application in thoracoscopic mediastinal reconstruction has not been reported.

**CASE PRESENTATION:**

A 56-year-old man with Siewert type II esophagogastric junction cancer required a high intramediastinal anastomosis because a proximal submucosal tumor necessitated extended esophageal resection. After laparoscopic PG and lower esophagectomy, a seromuscular tunnel was created extracorporeally on the remnant stomach. The remnant stomach was elevated into the mediastinum, and thoracoscopic reconstruction was performed in the prone position. Following fixation of the remnant stomach to the esophagus and rotation of the esophagogastric axis, esophagogastrostomy was completed using hand-sewn sutures, followed by seromuscular roof closure over the anastomosis. Reconstruction was completed in 60 minutes. A contrast study on POD 5 demonstrated no anastomotic leakage or reflux. The patient resumed oral intake on POD 6 and was discharged on POD 11 without complications.

**CONCLUSIONS:**

Thoracoscopic reconstruction using the NFTT is feasible and may represent a practical and technically simplified alternative to thoracoscopic DFT in selected patients requiring high intramediastinal anastomosis.

## Abbreviations

DFT
: double-flap technique
EGJC
: esophagogastric junction cancer
NFTT
: non-flap tunnel technique
PG
: proximal gastrectomy

## INTRODUCTION

The incidence of EGJC has been increasing, and PG is widely performed as a function-preserving procedure.^1,2)^ However, reconstruction after PG remains controversial because of the risks of reflux esophagitis, anastomotic stenosis, and anastomotic leakage.

The DFT is an established anti-reflux reconstruction method and has been associated with a low incidence of anastomotic leakage.^3–5)^ These characteristics may be advantageous for mediastinal reconstruction, where anastomotic failure can result in severe complications.

When extended esophageal resection is required, the anastomosis may be located high within the lower mediastinum, making transhiatal reconstruction technically demanding. Although thoracoscopic DFT reconstruction has been reported, most reports remain limited to isolated cases.^6–8)^ We recently reported a case series demonstrating the technical feasibility of thoracoscopic intrathoracic DFT for EGJC.^9)^ However, DFT remains technically demanding because flap-related procedures add complexity to reconstruction.

Recently, Hayami et al. introduced the NFTT as a simplified alternative to DFT after minimally invasive PG, eliminating the need for flap creation and closure.^10)^ We herein describe thoracoscopic NFTT reconstruction performed in a patient with EGJC requiring a high intramediastinal anastomosis.

## CASE PRESENTATION

**Fig. 1:**
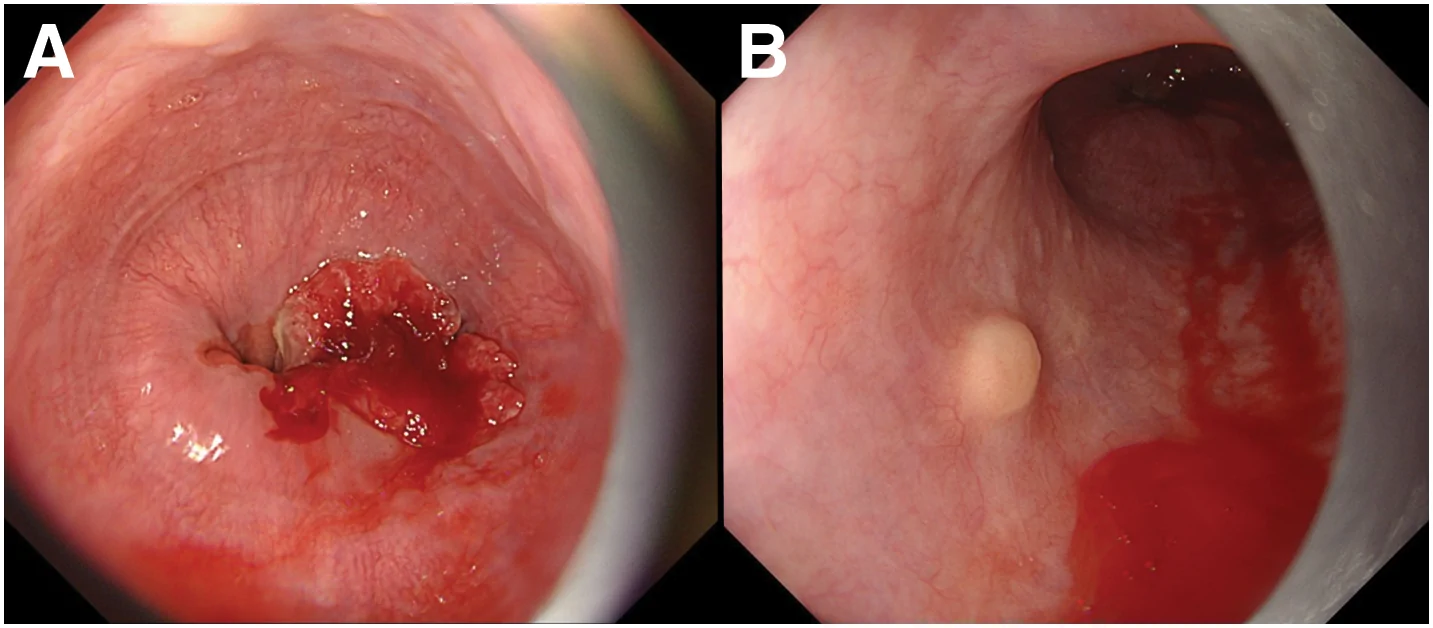
Upper gastrointestinal endoscopic findings. (**A**) Endoscopic examination revealed a Type 2 lesion at the esophagogastric junction with approximately 1 cm of esophageal invasion. (**B**) A submucosal tumor was identified approximately 4 cm proximal to the primary lesion, necessitating extended esophageal resection and a high intramediastinal anastomosis.

A 56-year-old man with no significant comorbidities was diagnosed with Siewert type II EGJC during routine screening. Endoscopic examination revealed a Type 2 lesion at the esophagogastric junction with approximately 1 cm of esophageal invasion. A submucosal tumor was also identified approximately 4 cm proximal to the primary lesion (**Fig. 1**). CT showed no lymph node or distant metastasis. The preoperative diagnosis was Siewert type II EGJC with well-differentiated adenocarcinoma, cT2N0M0, cStage I. Because an extended esophageal resection of approximately 5 cm was required to include the proximal submucosal tumor, a high intramediastinal anastomosis was necessary.

**Fig. 2:**
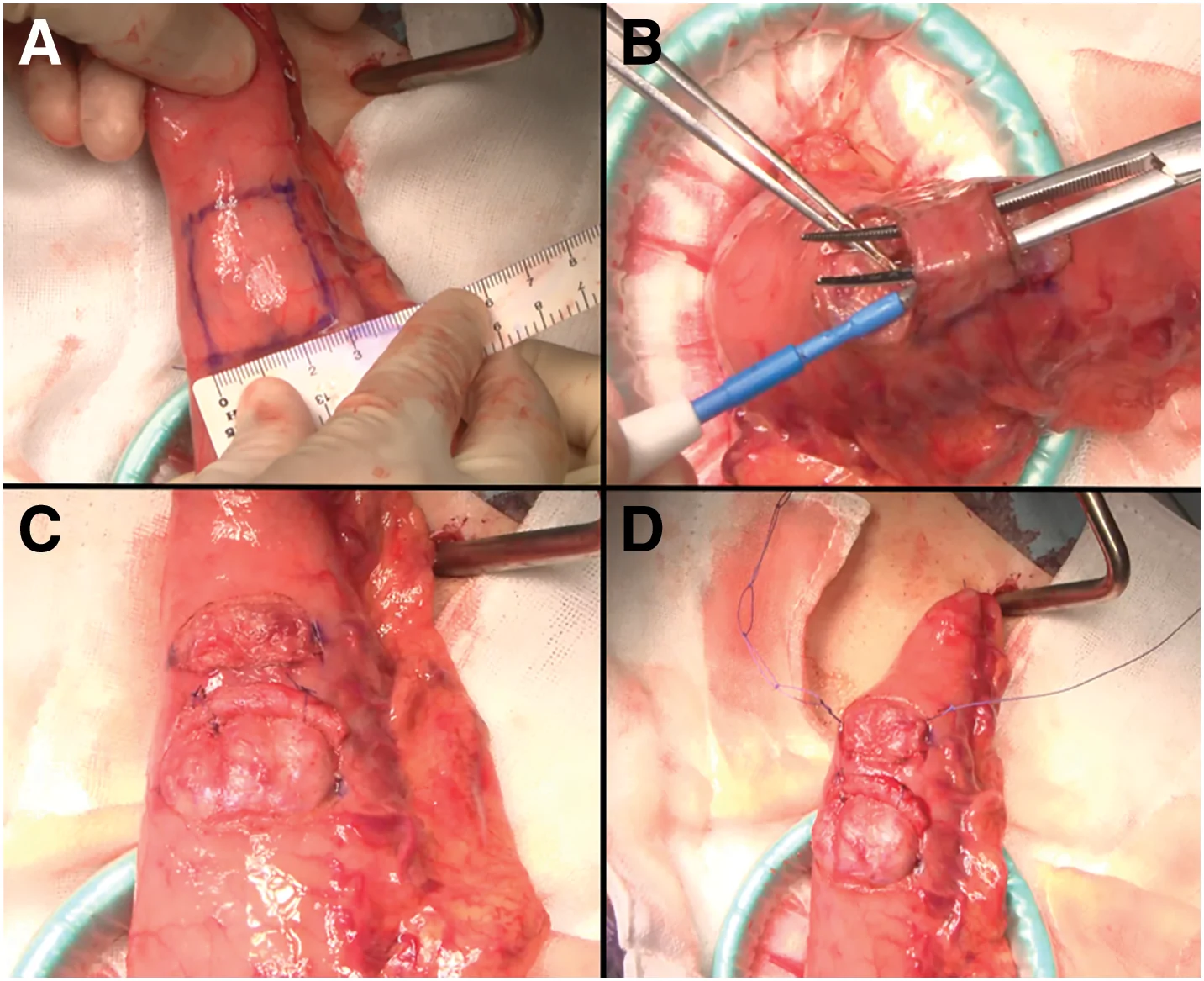
Extracorporeal creation of the seromuscular tunnel. (**A**) The planned seromuscular tunnel was marked at 2.5 cm in width and 3.5 cm in height. (**B**) A seromuscular tunnel with a roof was created on the anterior wall of the remnant stomach. (**C**) The inferior edge of the seromuscular roof was partially incised, rolled up, and temporarily secured to facilitate the subsequent anastomotic procedure. (**D**) A marking suture was placed at the proximal edge to maintain the orientation of the greater and lesser curvature sides and prevent torsion during thoracoscopic reconstruction.

The patient underwent laparoscopic PG and lower esophagectomy with lower mediastinal lymph node dissection. After completion of the abdominal procedure, a small epigastric incision was made, and the remnant stomach was delivered extracorporeally. A seromuscular tunnel with a roof measuring 2.5 cm in width and 3.5 cm in height was created on the anterior wall of the remnant stomach. To avoid torsion during thoracoscopic reconstruction in the prone position, the greater and lesser curvature sides were marked before elevation. The remnant stomach was then elevated into the mediastinum through the esophageal hiatus without torsion (**Fig. 2**).

**Fig. 3:**
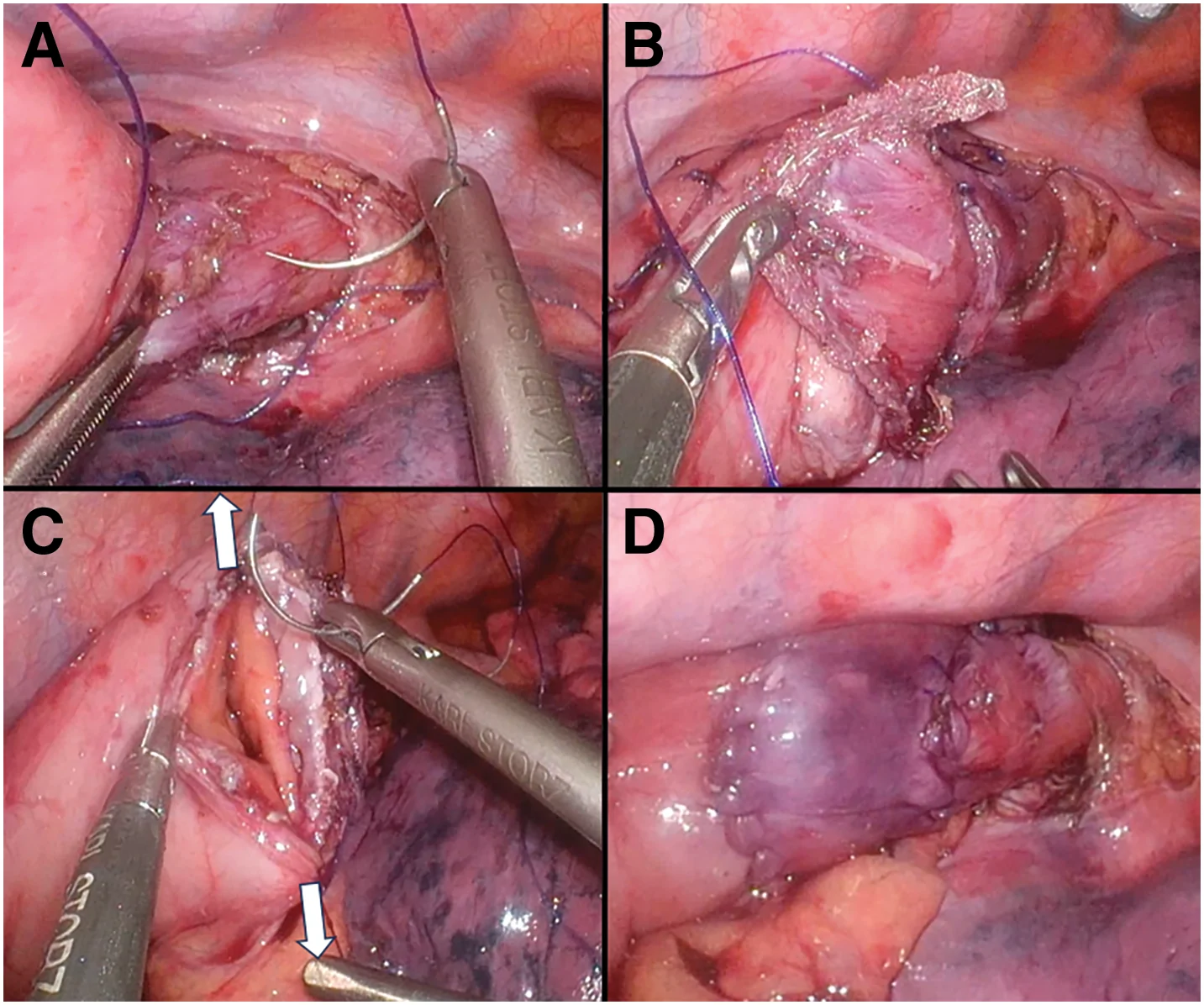
Thoracoscopic reconstruction through the right thoracic approach in the prone position. (**A**) The remnant stomach was fixed to the posterior wall of the esophagus using a single continuous barbed suture with 4 bites. (**B**) The esophageal stump was passed through the seromuscular tunnel and pulled caudally. (**C**) Both lateral edges of the anastomotic site were sutured, and the stay sutures were retracted by the assistant to achieve 90° rotation and obtain a direct operative view. (**D**) The seromuscular roof was repositioned over the anastomosis, and the cranial and caudal edges were continuously sutured to fully cover the anastomotic site.

The patient was placed in the prone position, and thoracoscopic reconstruction was performed through a right thoracic approach using 5 ports. The esophagus was mobilized sufficiently to achieve approximately 5 cm of length within the tunnel. The remnant stomach was fixed to the posterior wall of the esophagus using a single continuous barbed suture with 4 bites. After rotation of the esophagogastric axis, the anastomotic site was brought into a direct operative view. Esophagogastrostomy was performed using interrupted full-thickness sutures on the posterior wall to reduce the risk of anastomotic stenosis, followed by continuous barbed suturing of the anterior wall. The seromuscular roof was repositioned over the anastomosis, and its cranial and caudal edges were continuously sutured to fully cover the anastomotic site (**Fig. 3**). The key steps of thoracoscopic NFTT reconstruction are shown in **Supplementary Video 1**.

**Fig. 4:**
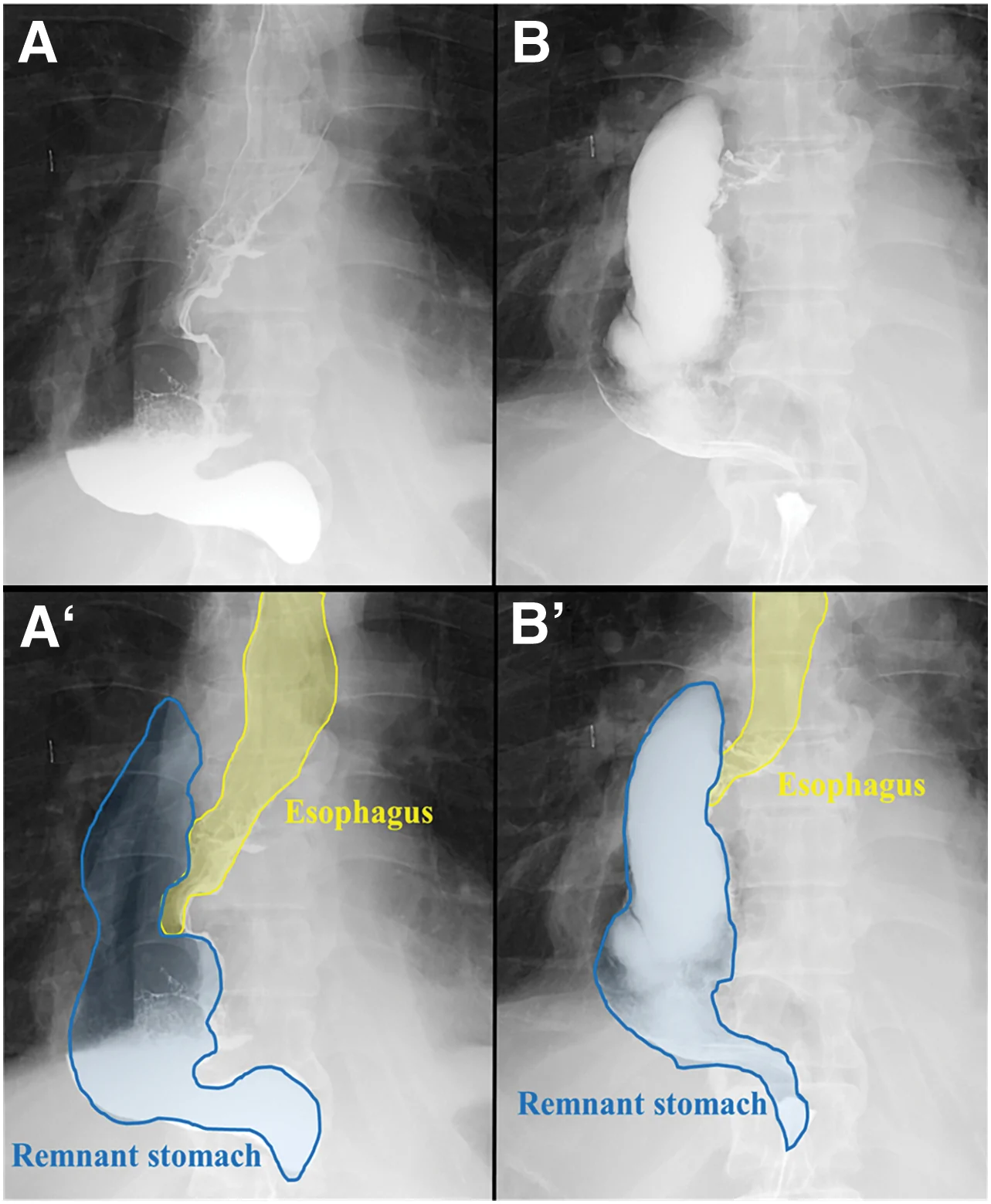
Postoperative contrast study findings. (**A**) The contrast agent passed smoothly through the anastomosis without leakage. (**B**) No reflux into the esophagus was observed in the Trendelenburg position. (**A**', **B**') Schematic tracings highlighting the relationship between the remnant stomach and the esophagus.

The total operative time was 386 minutes, including 60 minutes of thoracoscopic suturing for reconstruction, and blood loss was 1 mL. A contrast study on POD 5 confirmed the absence of anastomotic leakage and demonstrated smooth passage of contrast without reflux into the esophagus, even in the Trendelenburg position (**Fig. 4**). Oral intake was resumed on POD 6, and the patient was discharged on POD 11 without complications.

Histopathological examination revealed a well-differentiated adenocarcinoma with pT1b submucosal invasion and 1.5 cm of esophageal invasion without nodal or distant metastasis (pStage IA). Surgical margins were negative. A submucosal lesion located approximately 3.5 cm proximal to the main tumor was confirmed as leiomyoma on pathological examination.

## DISCUSSION

Thoracoscopic reconstruction with the NFTT was successfully performed after PG and lower esophagectomy for EGJC in a case requiring a high intramediastinal anastomosis. To our knowledge, this is the first report of thoracoscopic NFTT reconstruction for EGJC. A high intramediastinal anastomosis may be required when extended esophageal resection places the anastomotic site deep within the lower mediastinum, such as in patients with extensive esophageal invasion or concomitant proximal lesions requiring additional esophageal resection, as in the present case. Transhiatal reconstruction in these situations is technically challenging because the anastomosis must be performed in a narrow and deep operative field adjacent to critical structures. In contrast, thoracoscopic reconstruction provides a spacious and stable operative field, allowing safer and more precise suturing.

The DFT is an established anti-reflux reconstruction method and has been associated with a low incidence of anastomotic leakage in conventional reconstruction after PG.^3–5)^ These characteristics may be particularly advantageous in mediastinal reconstruction, where negative intrathoracic pressure may exacerbate reflux and anastomotic failure can result in severe complications. Based on this rationale, we previously reported the technical feasibility of thoracoscopic intrathoracic DFT for EGJC.^9)^ However, DFT remains technically demanding because flap-related procedures, including flap creation and closure, add complexity to reconstruction.

Hayami et al. recently introduced NFTT as a simplified alternative to DFT for minimally invasive PG. By eliminating seromuscular flap creation and closure, NFTT reduced reconstruction time and the incidence of anastomotic stenosis while maintaining anti-reflux efficacy.^10)^ These findings suggest that NFTT may simplify esophagogastrostomy without compromising its anti-reflux mechanism. These technical advantages may be particularly valuable during thoracoscopic reconstruction for a high intramediastinal anastomosis because NFTT eliminates flap-related procedures while preserving the anti-reflux concept of DFT.

Several technical strategies contributed to the successful and stable thoracoscopic NFTT reconstruction in this case. Marking of the greater and lesser curvature sides before elevation helped maintain orientation after transhiatal elevation of the remnant stomach. Fixation of the posterior esophageal wall to the remnant stomach followed by rotation of the esophagogastric axis provided a direct operative view of the anastomotic site. In addition, assistant retraction of the stay sutures helped maintain stable exposure throughout the anastomotic procedure. A potential concern associated with thoracoscopic intramediastinal reconstruction is a postoperative hiatal hernia or delayed gastric emptying caused by kinking of the remnant stomach. Post-esophagectomy hiatal hernia has been increasingly recognized after minimally invasive Ivor Lewis esophagectomy and may result in bowel incarceration requiring emergency surgery.^11,12)^ In the present case, neither complication occurred despite the absence of routine fixation at the esophageal hiatus. Nevertheless, after returning the patient to the supine position, gentle traction of the remnant stomach and fixation at the esophageal hiatus may be useful to minimize these risks.

In our previous thoracoscopic DFT series, the median reconstruction time was 78 minutes,^9)^ whereas reconstruction in the present NFTT case was completed in 60 minutes. Although a direct comparison is not possible, reconstruction in the present case was completed in a shorter time than that reported in our previous thoracoscopic DFT series.

## CONCLUSIONS

Thoracoscopic NFTT reconstruction may represent a practical reconstructive option for selected patients requiring a high intramediastinal anastomosis. Further studies are required to evaluate long-term functional outcomes, including reflux control and nutritional status.

## SUPPLEMENTARY MATERIALS

Supplementary Video 1

## DECLARATIONS

### Availability of data and materials

All data are included in this article.
